# Genetic inactivation of the vesicular glutamate transporter 2 (VGLUT2) in the mouse: What have we learnt about functional glutamatergic neurotransmission?

**DOI:** 10.3109/03009730903572073

**Published:** 2010-03-10

**Authors:** Åsa Wallén-Mackenzie, Hanna Wootz, Hillevi Englund

**Affiliations:** Department of Neuroscience, Unit of Developmental Genetics, Biomedical Center, Box 593, Uppsala University, S-751 24 UppsalaSweden

**Keywords:** Gene targeting, molecular biology, schizophrenia, glucose, amyotrophic lateral sclerosis, respiration

## Abstract

During the past decade, three proteins that possess the capability of packaging glutamate into presynaptic vesicles have been identified and characterized. These three vesicular glutamate transporters, VGLUT1–3, are encoded by solute carrier genes *Slc17a6–8*. VGLUT1 (*Slc17a7*) and VGLUT2 (*Slc17a6*) are expressed in glutamatergic neurons, while VGLUT3 (*Slc17a8*) is expressed in neurons classically defined by their use of another transmitter, such as acetylcholine and serotonin. As glutamate is both a ubiquitous amino acid and the most abundant neurotransmitter in the adult central nervous system, the discovery of the VGLUTs made it possible for the first time to identify and specifically target glutamatergic neurons. By molecular cloning techniques, different VGLUT isoforms have been genetically targeted in mice, creating models with alterations in their glutamatergic signalling. Glutamate signalling is essential for life, and its excitatory function is involved in almost every neuronal circuit. The importance of glutamatergic signalling was very obvious when studying full knockout models of both VGLUT1 and VGLUT2, none of which were compatible with normal life. While VGLUT1 full knockout mice die after weaning, VGLUT2 full knockout mice die immediately after birth. Many neurological diseases have been associated with altered glutamatergic signalling in different brain regions, which is why conditional knockout mice with abolished VGLUT-mediated signalling only in specific circuits may prove helpful in understanding molecular mechanisms behind such pathologies. We review the recent studies in which mouse genetics have been used to characterize the functional role of VGLUT2 in the central nervous system.

## Glutamate and vesicular glutamate transporters

**Figure 1.**
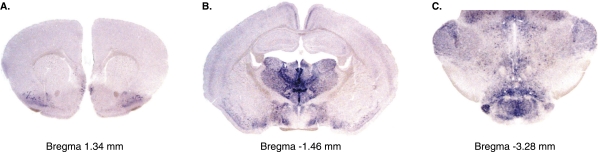
*In situ* hybridization for Vglut2 on free-floating coronal adult mouse brain sections showing the expression pattern at three different bregma levels. Vglut2 mRNA is detected by blue labelling and is seen in the piriform cortex in A (bregma 1.34 mm); in the thalamus, hypothalamus, piriform cortex, and retrosplenial group of the medial cortex in B (bregma −1.46 mm); and in many cell groups in the brain-stem, including the geniculate and mammillary nuclei in C (bregma −3.28 mm).

For 25 years, glutamate has been accepted as the most abundant excitatory neurotransmitter in the adult central nervous system (1), and glutamatergic neurons and glutamate-mediated excitatory signalling are implicated in all neuronal circuits of the central nervous system (2). Glutamate is necessary for brain function, and several diseases have been associated with defective or altered glutamate signalling, including Parkinson's disease, schizophrenia, Alzheimer's disease, amyotrophic lateral sclerosis (ALS), and depression (3–5). Increased knowledge of glutamatergic neurotransmission should therefore prove helpful in the treatment of several such disorders. As glutamate is a ubiquitous amino acid, the study of glutamatergic neurotransmission was for a long time hampered by the lack of markers specific for glutamate signalling neurons. Most of the current knowledge of glutamatergic neurotransmission is derived from pharmacological studies and gene targeting studies of the postsynaptic machinery, including both the cytoplasmic membrane transporters and the different kinds of glutamate receptors. However, none of these studies have directly addressed the role of the presynaptic glutamate-signalling neuron itself. A fundamental step forward for the research field around glutamate came with the recent identification that three members of the solute carrier family, *Slc17a6–8*, act as vesicular glutamate transporters (and were hence named VGLUT1, 2, 3). Their discovery has enabled proper identification of glutamate signalling neurons and studies of glutamatergic neurotransmission (6–8). VGLUT1 and VGLUT2 are considered the most reliable markers for glutamatergic neurons and represent important targets for the study of excitatory neurons *in vivo*. Their expression pattern is to a large extent complementary, with VGLUT1 mainly expressed in the cerebral and cerebellar cortex and hippocampus, and VGLUT2 mainly expressed in deeper brain regions including the thalamus and the brainstem (for an example of Vglut2 mRNA expression, see Figure 1, which was produced in our laboratory by digoxigenin-labelled *in situ* hybridization on vibratome-sliced sections) (7,9,10). The spatial distribution is not absolute, however; VGLUT2 is for example expressed in subpopulations of the cerebral cortex and hippocampus throughout life. There is also a temporal difference in VGLUT isoform expression, where VGLUT2 is most abundantly expressed during embryonic and early postnatal development, whereafter VGLUT1 becomes the dominating isoform in certain brain areas (11).

With the identification of the VGLUTs, it became possible to genetically alter glutamate signalling in the entire brain, or in specific brain circuits, and study the phenotype of these alterations *in vivo*. For example, the switch from VGLUT2 to VGLUT1 expression was observed *in vivo* by both Fremeau *et al*. and Wojcik *et al*. in 2004, when they genetically deleted VGLUT1 expression in mice using full knockout strategies (12,13). The *Vglut1^-/-^* mice survived 2 weeks after birth, but as the VGLUT1 expression increased during the third postnatal week in the wild-type animals, the *Vglut1^-/-^* mice started to die. These studies showed that VGLUT1 is not essential for life-supporting mechanisms immediately *ex utero*, a finding that was received with a certain degree of surprise (14). That glutamatergic signalling is required for new-born life was without doubt, and it was speculated that VGLUT2 may be the essential VGLUT. VGLUT2 is indeed expressed in several brainstem nuclei involved in control of both respiration and cardiovascular function (15,16) and is thereby heavily implicated in life-supporting mechanisms. However, VGLUT2 is broadly expressed in the brain and spinal cord and is likely to play various roles in many different neuronal circuitries, roles which are now, with the use of gene targeting, becoming increasingly known. Here we review the recent studies of VGLUT2 mouse genetics, a research field which is fairly new but which has already implicated VGLUT2 in diverse functions, some which were to a certain degree expected and some a bit more unexpected.

## Knock-out strategies

**Figure 2.**
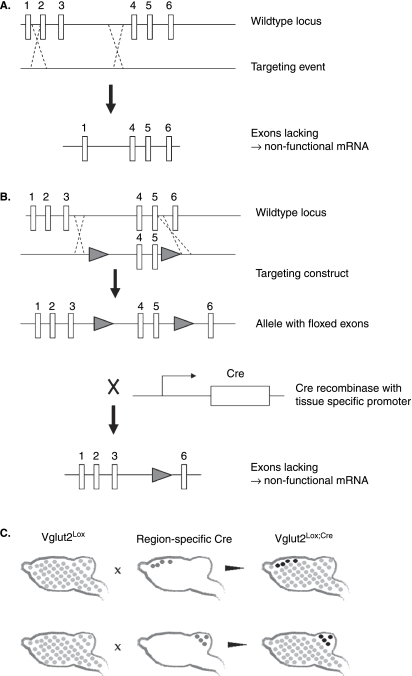
A: Full knockout models can be generated by homologous recombination between the wild-type allele and a targeting construct. The targeting construct lacks one or more exons of the gene, usually in combination with some selection sites. The result after homologous recombination is an allele lacking one or more exons, and which will produce a non-functional mRNA. B: Conditional knockouts are made in several steps, where first a targeting construct containing one LoxP site on each side of the exons to be deleted is combined with the wild-type locus through homologous recombination. Second, when mice carrying the allele with ‘floxed’ (i.e. flanked by LoxP sites) exons are crossed with mice expressing the Cre recombinase, the floxed exons are deleted resulting in a gene producing non-functional mRNA. C: Depending on which promoter that drives the Cre expression, the floxed gene—in this case *Vglut2*—can be deleted in specific tissues only. As schematically illustrated here, Vglut2lox mice (floxed Vglut2 in all cells is illustrated with bright grey dots) mated with Cre mice, where Cre is driven by forebrain or cerebellum-specific promoters (dark grey dots in upper and lower panels, respectively) will result in different conditional knockout mice where Vglut2 expression is specifically deleted in the Cre-expressing regions (illustrated by black dots).

Molecular biology and cloning strategies have enabled the possibility to unravel the role of VGLUT2-dependent glutamate signalling in specific neuronal circuits. In the research field of VGLUT2 mouse genetics so far, both full gene targeting (in which the gene is dysfunctional from start) and conditional gene targeting (in which the gene can be inactivated in a spatio-temporally restricted manner using the so-called Cre/LoxP system) have been utilized. We describe these techniques briefly in Figure 2. For a more extensive review on the Cre/LoxP system, see Nagy's excellent publication (17). We have chosen to present the VGLUT2 mouse genetic studies in an order related to the extent of the deletion, i.e. full knockout studies come first, followed by conditional knockout studies, in which the deletion is complete in specifically selected neurons, and lastly heterozygote studies are presented, in which the entire organism has a reduction, but not complete deletion, of VGLUT2 expression.

## Full knockout studies

In 2006, the first two studies of VGLUT2 gene targeting in the mouse were published. Our group, as well as that of Moechars and co-workers, both made full knockout studies, although by using different gene targeting strategies. Moechars *et al*. produced a mouse model where VGLUT2-dependent glutamate signalling was inhibited by a complete targeted deletion of the *Vglut2* gene (18). This deletion was made by the knockout strategy described in Figure 1A, where exon 2 was deleted by homologous recombination, leading to a disrupted open reading frame and a premature stop codon. Mice homozygous for the *Vglut2* deletion were, contrary to the *Vglut1*^-/-^, not viable at birth, and Western blot analysis of brain homogenates revealed a complete lack of VGLUT2 protein, while quantitative reverse transcriptase-PCR (RT-PCR) showed some residual VGLUT2 mRNA expression. Interestingly, this complete knockout of VGLUT2 was not compensated for by an increase in VGLUT1 levels, also shown by Western blot analysis. As the distribution of VGLUT1 and 2 differs between brain regions, the authors performed electrophysiological studies on cells isolated both from the hippocampus (with high VGLUT1 expression) and the thalamus (with high VGLUT2 expression) from wild-type and *Vglut2*^-/-^ mice respectively. These studies revealed a 95% reduction in evoked glutamate response in thalamic neurons from *Vglut2*^-/-^ mice, while hippocampal neurons functioned normally. Since the homozygotes were not viable, behavioural analyses were performed on mice heterozygous for the VGLUT2 deletion (*Vglut2*^+/-^). These studies are described in the section for heterozygote studies below.

In our case, the loss of VGLUT2 was achieved through a conditional approach using the Cre/LoxP system (19). We generated our *Vglut2^flox/flox^* mice by insertion of one LoxP site upstream of exon 4 of the mouse *Vglut2* genomic sequence and one LoxP site downstream of exon 6. Full knockout mice were obtained by mating of the *Vglut2^flox/flox^* mice with phosphoglycerate kinase I promoter (PGK)-Cre mice (20). None of the full knockout mice survived after birth, and the cyanotic appearance of the mice prompted us to focus on respiratory activities. Histological analyses revealed a complete lack of VGLUT2 protein but no gross malformations in the brain. However, lung alveoli were reduced in the *Vglut2^flox/flox;PGK-Cre^* mice as if non-inflated. When fetuses were surgically delivered at embryonic day 18.5 (E18.5), wild-type and heterozygote siblings immediately initiated breathing and showed a stable breathing behaviour. In contrast, no respiratory behaviour was observed in the full knockout embryos.

The respiratory central pattern generator (CPG) belongs to the better characterized CPGs, although before the production of VGLUT2 knockout mice it had been somewhat difficult to pin-point the role of glutamate signalling in this circuit. Since no respiratory behaviour was observed in the *Vglut2^flox/flox;PCre^* mice, we were interested in further investigating the respiratory CPG and in particular the pre-Bötzinger complex (PBC) which is a network pacing the respiratory rhythm. The PBC is located in the rostroventrolateral medulla, and its neurons are dependent on activation of glutamate receptors (N-methyl D-asperate (NMDA) and non-NMDA) to depolarize. By the targeted deletion of VGLUT2, we abolished the presynaptic release of glutamate and could show that this release was crucial to initiate the respiratory-related rhythm generation of the PBC. When fetuses were placed in a plethysmographic chamber to record ventilation, none of the *Vglut2^flox/flox;PCre^* showed any breathing behaviour, consistent with their malformed, defective lungs, while wild-type mice breathed at a normal frequency. To resolve the mechanisms behind this failed breathing initiation, we performed electrophysiology studies of brainstems from surgically delivered embryos. In contrast to wild-type preparations, recordings on brainstems from mutants showed no spontaneous rhythmic activity of the hypoglossal nerve root (12n). To investigate rhythm generation from the PBC, medullar slices were prepared and analysed through calcium imaging and extracellular electrophysiological recordings. For wild-type slices from E16.5 embryos, a spontaneous rhythmic activity was observed as changes in calcium-induced fluorescence, which as previously observed by others (21) could be reversed by blocking α-amino-3-hydroxyl-5-methyl-4-isoxazole-propionate (AMPA)/kainate receptors. However, for slices from the *Vglut2^flox/flox;PCre^*, no rhythmic activity could be observed. On a single-cell level, the fast AMPA/kainite-mediated synaptic events recorded in wild-type cells by whole-cell voltage clamp experiments were not observed in *Vglut2^flox/flox;PCre^* cells, suggesting that the lack of VGLUT2 mediates a selective absence of such fast synaptic events. Interestingly, thorough morphological and histological studies revealed no effect on the structural and cellular organization of the brainstem nuclei involved in respiration. Electron microscopy analyses of synaptic vesicles in *Vglut2^flox/flox;PCre^* mice showed a malformation of vesicles in asymmetric synapses in areas ‘normally’ rich in VGLUT2, though vesicles at symmetric synapses appeared morphologically normal. Taken together, by genetically eliminating VGLUT2 in mice, we could demonstrate the crucial need of VGLUT2-mediated glutamatergic signalling within the PBC to initiate respiratory behaviour already at the embryonic state. We also analysed the locomotor CPG, which has been suggested to be dependent on glutamatergic signalling mediated by VGLUT2 (22–25); however, we could not find evidence that this is the case. Thus, the respiratory CPG and the locomotor CPG do not seem to have a VGLUT2-dependency in common.

## Conditional knockout studies

### The hypothalamus

The first study using a cell type-specific Cre to delete VGLUT2 function was published by Tong and collaborators in 2007 (26). These authors generated *Vglut2^flox/flox^* mice by flanking exon 2 of the mouse *Vglut2* genomic sequence with LoxP sites, which resulted in a null allele as exon 1 only contains about 5% of the coding sequence of *Vglut2* and as the reading frame is disrupted. The authors were interested in evaluating the role of VGLUT2 in ventromedial hypothalamic (VMH) neurons, which are predominantly glutamatergic and previously were shown to express VGLUT2 (27). The VMH is believed to play a role in the neurocircuitry important for detection of hypoglycaemia and the subsequent counterregulatory responses that are activated to ensure that the brain receives adequate glucose supply. The counterregulatory measures that occur upon low blood glucose levels are the following: insulin secretion, glucagon secretion, epinephrine secretion (if needed), and, lastly, stimulation of food intake. While insulin secretion is believed to be primarily a response of pancreatic beta cells, the other three responses are primarily driven by the brain. The exact nature of the neurocircuitry underlying these responses and the mechanisms of its function are not known but are important to unravel, not least as the counterregulatory responses are dysfunctional in patients that suffer from diabetes.

The brain contains two kinds of glucose-sensing neurons: ‘glucose-excited’ neurons that increase their firing rate as glucose levels rise, and ‘glucose-inhibited’ neurons that decrease their firing as glucose levels fall. The decreased activity of the ‘glucose-excited’ neurons, the increased activity of the ‘glucose-inhibited’ neurons action, input from peripheral blood glucose sensors, as well as a combination of the three, are suggested as the means by which the brain detects low glucose levels. The glucose-sensing neurons are abundant in parts of the hypothalamus, including the VMH, the lateral hypothalamus, and the arcuate nucleus, and in the hindbrain. The presence of glucose-sensing neurons implicates the VMH in the neurocircuitry behind detection of hypoglycaemia and the adequate counterresponses. Tong and collaborators set out to test directly the hypothesis that the glutamatergic neurons of the VMH play a role in this neurocircuitry by inactivating VGLUT2 specifically in these neurons. For this, they crossed their SF-1-Cre transgenic mice (28), which express Cre exclusively in the VMH, to their *Vglut2^flox/flox^* mice and analysed the offspring. In their first experiment, they performed electrophysiological recordings on autaptic cultures of steroidogenic factor-1 (SF-1)neurons and could show two findings: 1) most of the SF-1 neurons are glutamatergic while a smaller proportion is GABAergic, and 2) glutamate release is disrupted in their *Vglut2^flox/flox;SF1-Cre^* mice (no excitatory postsynaptic currents (EPSCs) were detected in the knockout neurons). Having confirmed this, the authors analysed features related to feeding and blood glucose levels. Tong *et al*. found that the knockout mice had normal body-weight when fed a standard chow diet but developed some increased body-weight compared to controls when fed a high-fat, high-sucrose diet. The authors concluded that release of glutamate from the SF-1 neurons plays a small role in regulating energy balance upon fat-feeding but not in standard feeding.

The mice were then subjected to fasting for 24 hours, whereupon blood glucose, insulin, and glucagon were measured. The findings were quite striking. While insulin levels were similar in the knockout and control groups, blood glucose and glucagon levels were lower in the knockouts. The pancreatic hormone glucagon works on the liver and stimulates break-down of glycogen to glucose (glycogenolysis) as well as synthesis of glucose from other sources (gluconeogenesis). Gluconeogenesis can be detected in liver-derived mRNA as it leads to transcriptional regulation of gluconeogenic enzymes and a co-activator, PGC-1α. While these liver-expressed genes were transcriptionally increased in the controls, no increased gene expression was observed in the knockouts. These experiments showed that the impaired glucose homeostasis in the knockout mice was mediated by failure to increase blood glucagon levels which was followed by failure to induce expression of gluconeogenic genes in the liver.

An acute injection of insulin revealed that the *Vglut2^flox/flox;SF1-Cre^* mice suffered a more substantial fall in blood glucose than did the controls, a greater degree of hypoglycaemia that was followed by impaired glucagon response. The authors then performed hypoglycaemic clamp studies, where hypoglycaemia was induced by infusion of human regular insulin and sustained at similar levels in a controlled way for 60–90 minutes during which samples for epinephrine and glucagon were taken. This analysis showed that the knockout mice required higher rates of glucose infusion to keep the clamp, a finding consistent with decreased rates of glucagon production, which was also detected. In addition, a hypoglycaemia-induced increase in epinephrine observed in the controls was not detected in the knockout mice. The authors then treated the mice with 2-deoxyglucose (2-DG) by injection into the third ventricle, which in the controls led to brain-mediated increased blood glucose levels, plasma glucagon levels, and increased gene expression of gluconeogenic genes in the liver. None of these responses were observed in the *Vglut2^flox/flox;SF1-Cre^* mice. Together, these experiments showed that the counterregulatory responses to hypoglycaemia are deficient in the *Vglut2^flox/flox;SF1-Cre^* mice, and due to the very selective targeting event of *Vglut2* in this mouse model the authors were able to confidently suggest that the effects are mediated via loss of glutamate release in the VMH.

### The cortex and amygdala

In the beginning of 2009, we published our first paper where we had used a region-specific Cre mouse to delete VGLUT2 function in specific neurons (29). Based on gene expression analysis, it is clear that the majority of neurons in the postnatal cortex and hippocampus predominantly express VGLUT1 (7,9,10,15,30–32). However, a few cells in discrete regions of the cortex, such as in the retrosplenial group and the piriform cortex, as well as neurons in layers III and V/VI, do express certain levels of VGLUT2, as does the subiculum of the hippocampus. We were interested in analysing the functional role of these neurons and used the CamKII-Cre mouse to target these neurons in our *Vglut2^flox/flox^* mice. The CamKII-Cre mouse has been carefully analysed and the Cre recombinase has been shown to be expressed in the cortex, hippocampus, striatum, and amygdala (33). VGLUT2 is not expressed in the striatum, but is expressed in a subset of the amygdaloid nuclei. Although we had not anticipated a major phenotype, it became evident when ear-tagging the first litters of *Vglut2^flox/flox;CamKII-Cre^* mice after weaning that they had a clearly noticeable behaviour, in that the mice moved around a lot and instead of trying to avoid the caretaker's hand rather turned towards the hand. After having confirmed the loss of Vglut2 mRNA in the expected cortical areas and amygdaloid areas and a subsequent loss of VGLUT2 protein in target areas of these neurons, we set out to analyse the behaviour of the mice using validated behavioural paradigms. Based on our initial observations, we first analysed anxiety-related behaviour in the Elevated Plus Maze (EPM) and found an increased frequency and duration in the open arm, especially in the outer part of this arm, in the knockouts compared to the controls. In addition to suggesting a decreased anxiety-related behaviour, analysis of the total frequency in the different compartments showed that the knockout mice were significantly more active than the controls. We then turned to the more sensitive Multi-Concentric Square Field (MCSF) (34), developed by Erika Roman and Bengt Meyerson at the Biomedical Center in Uppsala, in which the mice are allowed freely to explore a challenging environment. In addition to confirming the hyperactivity of the *Vglut2^flox/flox;CamKII-Cre^* mice, the MCSF also revealed that the knockout mice to a lesser extent avoided the open area and that they spent less time in the dark corner and more time in areas associated with risk assessment and risk-taking. The altered emotional behaviour observed in the EPM and MCSF was followed by social behaviour analyses to assess the social competence of the mice. The *Vglut2^flox/flox;CamKII-Cre^* mice showed increased dominance but also increased interaction with their counterparts. We also analysed cognitive functions by using the Morris Water Maze. While showing no defects in the learning process using this paradigm, the *Vglut2^flox/flox;CamKII-Cre^* mice did display a reduction in spatial memory function, when a probe test was performed 6 days after the last training day. Cognitive deficiency, such as decreased memory function, is one criterion for schizophrenia, a human disorder also characterized by positive symptoms such as hallucinations and delusions, and negative symptoms such as blunted emotional expression and altered social behaviour. While hallucinations and delusions cannot be analysed in animal models of this disease, hyperactive behaviour has been classified as a positive symptom of schizophrenia-like behaviour. Positive and negative symptoms together with cognitive deficiency are also criteria for schizophrenia-like behaviour in animal models of the disease. The *Vglut2^flox/flox;CamKII-Cre^* mice fulfil all these three groups of criteria for schizophrenia-like behaviour. In addition, the *Vglut2^flox/flox;CamKII-Cre^* mice display altered prepulse inhibition as well as an effect on dopaminergic signalling, features considered as cardinal symptoms of schizophrenia and schizophrenia-like behaviour. We suggest that the *Vglut2^flox/flox;CamKII-Cre^* mice may function as a genetic hypoglutamate model of schizophrenia-like behaviour and may be of use for validation of new psychopharmacological drugs.

## Heterozygote studies

The *Vglut2^-/-^* mice are models of completely absent VGLUT2-mediated glutamate signalling, either in the whole brain or in specific neuronal circuits. There is evidence that alterations in the number of VGLUT2 molecules also affect the glutamate signalling properties (18,35,36) and that such an alteration in both gene expression and protein levels are associated with disease (5,37–39). One theory is that the number of VGLUT molecules available at the presynapse is in direct correlation with the amount of glutamate packaged into the vesicles, which in turn affects the efficiency of the signalling itself. This dose-dependence can be studied using heterozygous knockout mice, having their VGLUT2 expression reduced by approximately 50%. Moechars and co-workers used their full knockout model, described above, for such studies and observed through electrophysiological studies on cultured neurons that cells from homozygous mice had almost no residual excitatory transmission, while cells from heterozygous mice had a functional signalling, although reduced by 25% compared to wild type, measured as the postsynaptic response to one single vesicle fusion—the quantal size. The quantal size varies depending on several parameters, including concentration of glutamate in the vesicle (40), which is why a reduction in the number of VGLUT2 proteins is likely to cause the reduced postsynaptic response by less glutamate transport into the vesicles and thus a reduced filling state. Conversely, over-expression of VGLUTs has been demonstrated to increase quantal size (36).

### Thalamus and pain

To investigate the phenotype caused by the 50% reduction in VGLUT2 protein levels in heterozygotes (18), Moechars *et al*. compared *Vglut2*^+/-^ to wild-type mice in a battery of behavioural tests. While there was no difference between the genotypes in motor function, learning and memory, acute nociception, or inflammatory pain, they had alterations in tests indicative of neuropathic pain. Acute nociception was unchanged in *Vglut2*^+/-^ mice, which indicates that the glutamatergic signalling in the nociceptive pathway is independent of VGLUT2, unaffected by the 50% decrease in VGLUT2 levels, or compensated by an increased peripheral VGLUT1 expression. The same seems to be the case for inflammatory pain, as tested by the formalin test which involves glutamatergic sensitization in the periphery and spinal cord. According to the authors, the reduction of neuropathic pain in heterozygous mice supports the involvement of VGLUT2-dependent glutamate signalling from the thalamus in such responses (18).

The same model was further studied by the same group, and it was compared to heterozygous *Vglut1* knockout mice in a study using additional behavioural pain analyses (41). *Vglut1^+/-^* mice displayed no alterations in neuropathic pain tests, whereas *Vglut2^+/-^* did also in this study. Here, two additional tests of nerve injury were included—spared nerve injury (SNI) and chronic constriction injury (CCI). After surgery, mice were tested using the von Frey set-up to assess mechanical sensitivity and the writhing test with acetone exposure to assay for chemical nociception. All mice showed a lowered threshold in von Frey's as a response to the mechanical allodynia caused by the SNI, except the *Vglut2^+/-^* mice which did not respond to the injury. The same was observed after acetone exposure, where *Vglut2^+/-^* mice failed to develop a cold allodynia-like response to injury. For the CCI test of mechanical allodynia, *Vglut2^+/-^* mice did not differ from controls in von Frey's test but did respond to acetone spray, however to a lesser extent than did wild-type mice. Thus, *Vglut2^+/-^* mice fail to display typical signs of neuropathic pain. Both these studies, where different aspects of pain were investigated, converge on VGLUT2-dependent signalling—and not VGLUT1—as being an important player in mediating neuropathic pain responses, a finding of importance not least for drug development in the pain area.

### Hippocampus and epilepsy

Alterations in glutamatergic signalling has, as described above, been implicated in several diseases and among them epilepsy. For example, studies in Mongolian gerbils demonstrated an increased VGLUT2 immunoreactivity in parts of the hippocampus in epileptic seizure-prone gerbils (42), which could be related to a hyperexcitability in these animals. These studies prompted Schallier and co-workers to study epileptic-like behaviour on their *Vglut2^+/-^* mice (43). Epileptic seizures can be induced experimentally by administration of pentylenetetrazol (PTZ), which in rodents induces different types of seizures at different doses. The seizures can be recorded in electroencephalograms (EEGs). *Vglut2^+/-^* mice, from the same mouse line as used for the pain studies described above (18,41), needed lower doses of PTZ than did wild-type litter-mates to induce the first myoclonic twitch as well as the fore-limb clonus, while the threshold dose for tonic hind limb extension and the lethal dose did not differ significantly between groups. Importantly, the *Vglut2^+/-^* mice had no alterations in base-line EEG, and thus their 50% reduction in VGLUT2 levels did not seem to induce spontaneous seizures. This enhanced sensitivity to clonic epileptic seizures in *Vglut2^+/-^* mice could, according to Schallier *et al*., be explained by alterations in the thalamocortical circuitry caused by their 50% reduction of VGLUT2 in e.g. thalamus. Projections going from thalamus to the cortex are mainly glutamatergic, and this pathway also sends collateral excitatory projections to the reticular thalamic nucleus (RTN). The neurons of RTN then send inhibitory GABAergic signals back to the thalamocortical neurons. These neurons are also intensively activated by PTZ (44). In *Vglut2^+/-^* mice this leads to an increased inhibition from the RTN, which further decreases the excitatory output of the thalamocortical neurons. According to the authors, this altered signalling could explain their enhanced sensitivity to PTZ.

### Motor neurons and amyotrophic lateral sclerosis

Glutamate is the most abundant excitatory neurotransmitter, and its presence in the extracellular space must be tightly regulated as excessive release of glutamate into the synaptic cleft can induce excitotoxicity and death of the postsynaptic neuron. This excitotoxicity has been suggested to be involved in the pathogenesis of amyotrophic lateral sclerosis (ALS), a disease where motor neurons gradually die leading to paralysis of the patient. The only approved drug to treat ALS reduces excitotoxicity by reducing glutamatergic signalling (45). A decrease in glutamatergic signalling could prove beneficial for survival of motor neurons and halting the progression of ALS. To test this hypothesis, we crossed our *Vglut2^flox/+^*mice with a *Sod1^G93A^* mouse, a well characterized model for ALS which has previously been demonstrated to show an ALS-like phenotype with progressive loss of motor neurons. In this way, we generated an ALS mouse model with attenuated VGLUT2-dependent glutamatergic signalling (46). *Sod1^G93A^;Vglut2^flox/+^*mice did have reduced VGLUT2 protein levels in their spinal cord, not compensated by an increase in VGLUT1, but had no alterations in disease onset, life-span, or weight loss compared to *Sod1^G93A^;Vglut2^+/+^* litter-mate mice. However, immunohistochemical analyses of spinal cord sections revealed that a reduction in VGLUT2 levels increased viability of larger neurons—most likely motor neurons—of the spinal cord. In addition, expression studies of motor neuron-specific markers, vesicular acetylcholine transporter and the high affinity choline transporter, demonstrated that motor neurons in the lumbar spinal cord were partly spared from degeneration by reduced VGLUT2 expression in *Sod1^G93A^;Vglut2^flox/+^* mice. To investigate if these rescued neurons still were connected to the muscle we investigated neuromuscular junctions (NMJs) in tibialis anterior. In control mice, almost all NMJs were innervated by motor neurons, an innervation that was markedly reduced in the *Sod1^G93A^*;*Vglut2^flox/+^* mouse. Thus, in the *Sod1^G93A^*;*Vglut2^flox/+^* model, parts of the reduced NMJ innervation could be rescued. This indicated to us that the rescued motor neuron cell-bodies in the spinal cord were in fact connected to the muscle. Interestingly, cholinergic motor neurons in two cranial nuclei (VII and XII) of the brainstem responded differently to reduced VGLUT2. In the VIIth cranial nucleus, we observed a complete rescue of motor neurons by reducing VGLUT2 levels, while the XIIth cranial nucleus was completely unaffected by the disease and no cell loss was observed.

By creating this heterozygotic *Vglut2^flox/+^* mouse model we were able to evaluate a putative role for VGLUT2 in ALS. This study shows that glutamate excitotoxicity is part of the ALS disease progression and that different motor neuron populations have different susceptibility for this toxicity. Hence, decreased glutamate signalling could be beneficial for motor neuron survival in ALS.

**Table I.** A summary of the different Vglut2 inactivation events, the cell population they affect and the phenotypes they give rise to.

Type of Vglut2 inactivation	Cre-promoter	Name	Region affected	Major phenotype	Ref.
Full knockout, conditional strategy	PGK	*Vglut2^flox/flox;PCre^*	All cells	Lethal phenotype. No activity in respiratory CPG while normal activity of locomotor CPG	(19)
Conditional knockout	SF-1	*Sf1-Cre,Vglut2^flox/flox^*	VMH	Defective counterregulatory responses to hypoglycaemia	(26)
Conditional knockout	CamKII	*Vglut2^flox/flox;CaMKII-Cre^*	Cortex, amygdala	Hyperactivity, reduced memory and prepulse inhibition, schizophrenia-like behaviour. Effect on the dopamine system	(29)
Heterozygous full knockout, conditional strategy, crossed with ALS mouse model	PGK	*Sod1^G93A^;Vglut2^flox/+^*	All cells	Rescued motor neuron viability	(46)
Full knockout		*Vglut2^-/-^*	All cells	Lethal phenotype. No VGLUT2 protein expression and no up-regulation of VGLUT1	(18)
Heterozygous through full knockout strategy		*Vglut2^+/-^*	All cells	Impaired acquisition of neuropathic pain; Increased sensitivity to PTZ clonic seizures.	(41); (43)

CPG = central pattern generator, ALS = amyotrophic lateral sclerosis, VMH = ventromedial hypothalamus, PTZ = pentylenetetrazol

## Concluding remarks

During the past few years, several laboratories have used gene-targeting techniques in mice to study the functional roles of all three VGLUTs, i.e. VGLUT1–3. Our group has, together with others around the world, focused on VGLUT2, the predominant isoform during embryonic development and in deep brain structures, including the thalamus, in the adult. Full knockout analyses revealed that VGLUT2 is essential for life *ex utero*, as new-born pups die immediately after birth due to dysfunctional respiratory CPG. Using the conditional approach, VGLUT2 has been linked to normal function of the hypothalamic regulation of feeding and maybe somewhat more surprisingly, given the restricted distribution of VGLUT2 in the cerebral cortex, to higher brain function. These two studies, in which the Cre/LoxP system was used, have given novel insights into mechanisms that may be useful for the understanding of human pathologies, in these cases diabetes and schizophrenia-like behaviour. Also heterozygous VGLUT2 mice have been analysed with the purpose of addressing human conditions (both pain and epilepsy have been addressed), and it has been shown that VGLUT2 does play a role in mediating these conditions. Further analyses will reveal more about the mechanisms behind these findings. In a recent study, a connection between VGLUT2 gene expression and ALS was shown, which specified a role for VGLUT2 in motor neuron survival.

The research field of mouse genetics of the VGLUTs is fairly new, and we expect that the next few years will see a rapid development in this field, which will certainly give new insights into the role of the different VGLUTs in glutamatergic neurotransmission in the central nervous system.
